# Influenza-Associated Pediatric Deaths — United States, 2024–25 Influenza Season

**DOI:** 10.15585/mmwr.mm7436a2

**Published:** 2025-09-25

**Authors:** Katie Reinhart, Stacy Huang, Krista Kniss, Carrie Reed, Alicia Budd

**Affiliations:** 1 Influenza Division, National Center for Immunization and Respiratory Diseases, CDC.

**Summary**

**What is already known about this topic?**

Influenza can cause severe illness and death among all persons, including children.

**What is added by this report?**

The 2024–25 influenza season had the highest number of pediatric deaths reported (280) since child deaths became nationally notifiable in 2004, except for the 2009–10 influenza A(H1N1)pdm09 pandemic. Approximately one half of children who died from influenza had an underlying medical condition, and 89% were not fully vaccinated.

**What are the implications for public health practice?**

All persons aged ≥6 months who do not have contraindications should receive an annual influenza vaccination to prevent influenza and its complications, including influenza-associated death.

## Abstract

Influenza-associated deaths among children aged <18 years have been nationally notifiable since 2004. The highest number of pediatric deaths reported during a single season since reporting of influenza-associated pediatric deaths began (excluding the 2009–10 influenza A[H1N1]pmd09 pandemic) occurred during the 2024–25 season. Through September 13, 2025, a total of 280 influenza-associated pediatric deaths were reported, representing a national rate of 3.8 deaths per 1 million children. The median age at death was 7 years, and 56% of children who died had at least one underlying medical condition. Influenza A viruses were associated with 240 (86%) of the deaths. Forty percent of children who died were treated with influenza antiviral medications. Among the 208 pediatric decedents with available data who were eligible for influenza vaccine, 89% were not fully vaccinated. CDC recommends that all persons aged ≥6 months who do not have contraindications receive the influenza vaccine each year, ideally by the end of October.

## Introduction

Influenza can lead to severe illness and death. Vaccination against influenza is recommended for all persons aged ≥6 months who do not have contraindications, to prevent influenza and its associated complications (*1*). Some children are at higher risk for death from influenza based on their age, underlying medical conditions, and vaccination status.

Surveillance for pediatric influenza-associated mortality began in 2004, after reports of increased numbers of influenza-associated deaths among children (*2*). Since that time, the highest number of reported pediatric deaths (288) occurred during the 2009–10 influenza A(H1N1)pdm09 pandemic, and, until the current season, the second highest number (210) was reported during the 2023–24 season. During the 2020–21 season, when implementation of numerous strategies to prevent transmission of SARS-CoV-2 sharply reduced circulation of influenza viruses, only one influenza-associated death in a child was reported. This report describes influenza-associated pediatric deaths during the 2024–25 season.

## Methods

### Ascertainment of Influenza-Associated Pediatric Deaths

Data on influenza-associated deaths were obtained from the Influenza-AssociatedPediatricMortalitySurveillanceSystem. An influenza-associated pediatric death is defined as a death in a person aged <18 years, resulting from an influenza-compatible clinical illness, confirmed by an appropriate diagnostic test to be influenza, with no period of complete recovery between the illness and death (Influenza-associatedpediatricmortalityreport,CouncilofStateandTerritorialEpidemiologists). State and local health departments identify these deaths and report them to CDC using standardized case report forms.* Children who lived in the United States and who died during week 40 of 2024 through week 37 of 2025 (September 29, 2024–September 13, 2025) were included. The final case count might increase as additional reports are received. Population estimates of children aged <18 years were obtained from the UnitedStatesCensusBureau.

### Analysis

Variables associated with health, including underlying medical conditions, vaccination status, and health care use during illness are described. Children eligible for influenza vaccine and for whom case report forms contained sufficient information to determine vaccination status were categorized as either fully vaccinated or not fully vaccinated.^†^ SAS (version 9.4; SAS Institute) was used to perform all statistical analyses. This activity was reviewed by CDC, deemed not research, and was conducted consistent with applicable federal law and CDC policy.^§^

## Results

### Demographic Characteristics of Pediatric Influenza-Associated Deaths

During the 2024–25 influenza season, a total of 280 pediatric deaths were reported, representing a national rate of 3.8 deaths per 1 million children (Table 1). The median age at time of death was 7 years (IQR = 2–11 years); 61% of deaths occurred among children aged <9 years. The influenza-associated mortality rate was highest among children aged <6 months (11.1 per 1 million) and was higher among females (4.5) than males (3.1). White children accounted for the highest percentage of deaths (42%) but had the second lowest death rate (3.1) after Asian children (2.8). The highest mortality rate occurred among children who were Black or African American (5.8), who accounted for 23% of all pediatric influenza deaths. The number of influenza-associated pediatric deaths peaked at 28 during weeks 6 and 7 of 2025 (week ending February 8 and February 15) (Figure).

**TABLE 1:** Characteristics of children aged <18 years who died from influenza-associated illness and influenza-associated mortality, by selected demographic characteristics — United States, September 29, 2024–September 13, 2025

Characteristic	No. of deaths (%)	U.S. population, no.	Influenza death rate*
**Overall**	**280 (100)**	**73,132,720**	**3.8**
**Age group**			
**Median age group (IQR)**	7 (2–11)	—	—
<6 mos^†^	20 (7)	1,807,799	11.1
6–23 mos^§^	41 (15)	5,509,623	7.4
24–59 mos	48 (17)	11,281,892	4.3
5–8 yrs	62 (22)	16,024,708	3.9
9–12 yrs	53 (19)	16,614,665	3.2
13–17 yrs	56 (20)	21,894,033	2.6
**Sex**			
Female	161 (58)	35,727,465	4.5
Male	116 (42)	37,405,255	3.1
**Race and ethnicity^¶^**			
Asian	12 (5)	4,269,721	2.8
Black or African American	59 (23)	10,138,247	5.8
Hispanic or Latino	71 (28)	19,688,847	3.6
White	108 (42)	34,765,741	3.1
Other	8 (3)	—	—

* Influenza deaths per 1 million children aged <18 years.

^†^ The population estimate for age <6 months was calculated as the population of children aged <12 months divided by 2.

^§^ The population estimate for ages 6–23 months was calculated as the population of children aged <12 months divided by 2 plus the population of children aged 1 year.

^¶^ Categories with fewer than five deaths were combined into an “Other” category. Rates were not calculated for this category. All children with Hispanic or Latino (Hispanic) ethnicity reported were included in the Hispanic category and not included in the categories Asian, Black or African American, White, or Other.

**FIGURE:**
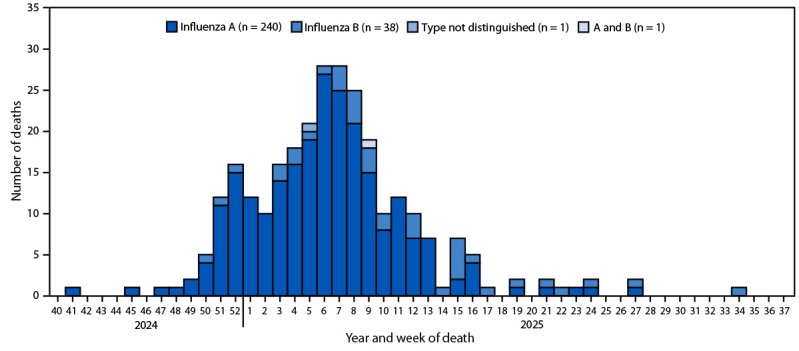
Influenza-associated deaths among children aged <18 years, by week and virus type (N = 280) — United States, September 29, 2024–September 13, 2025

### Influenza Virus Types

Reverse transcription–polymerase chain reaction (RT-PCR) testing was performed on specimens from 251 (90%) decedents; among the 29 children whose specimens did not undergo RT-PCR testing, specimens of 26 (90%) received rapid influenza testing, and three (10%) received viral culture testing. Among the 280 pediatric influenza-associated deaths, influenza A viruses were associated with 240 (86%) and influenza B viruses with 38 (14%) (Table 2). Among the 169 (70%) influenza A deaths with a known subtype, 95 (56%) were A(H1N1)pdm09 viruses, 73 (43%) were A(H3N2) viruses, and one (<1%) had both A(H1N1)pdm09 and A(H3N2) detected.

**TABLE 2:** Number and percentage of children aged <18 years who died from influenza-associated illness, by selected characteristics — United States, September 29, 2024–September 13, 2025

Characteristic	No. of deaths (%)
**Total deaths**	**280 (100)**
**PCR testing done**	
Yes	251 (90)
No	29 (10)
**Influenza virus type and subtype/lineage**	
Influenza A	240 (86)
A(H1N1)pdm09*	95 (56)
A(H3N2)*	73 (43)
A(H1N1)pdm09 and A(H3N2) co-infection*	1 (1)
Subtype not known	71 (—)
Influenza B	38 (14)
B Victoria^†^	4 (100)
Lineage testing not performed	34 (—)
A and B	1 (0)
A/B not distinguished	1 (0)
**ACIP-defined high-risk medical conditions^§^**	
Yes, any	148 (56)
No, none	114 (44)
Missing	18 (—)
**Number of ACIP-defined high-risk medical conditions^¶^**	
1	76 (51)
2	43 (29)
3	20 (14)
4	8 (5)
5	0 (0)
6	1 (1)
Type of medical conditions**	
Neurologic disorder	93 (35)
Moderate or severe developmental delay	59 (23)
Seizure disorder	46 (18)
Cerebral palsy	27 (10)
Neuromuscular disorder	22 (8)
Other neurologic disorder	51 (19)
Pulmonary disease	43 (16)
Asthma or reactive airway disease	28 (11)
Chronic pulmonary disease	16 (6)
Chromosome/genetic disorder	43 (16)
Congenital heart disease or other cardiac disease	30 (11)
Immunosuppressive condition	11 (4)
Received steroids before illness	3 (1)
Cancer (received chemotherapy or radiation)	3 (1)
Endocrine disorder	14 (5)
Diabetes mellitus	3 (1)
Obesity	9 (3)
Mitochondrial disorder	3 (1)
Renal disease	8 (3)
Pregnant	0 (—)
**Complications during acute illness**	
Yes	218 (88)
No	29 (12)
Unknown	33 (—)
**Complications^††^**	
Shock or sepsis	108 (50)
Pneumonia	82 (38)
Acute respiratory distress syndrome	60 (28)
Seizures	53 (24)
Encephalopathy/encephalitis	40 (18)
Cardiomyopathy/myocarditis	28 (13)
Bronchiolitis	11 (5)
Hemorrhagic pneumonia/pneumonitis	4 (2)
Croup	1 (0)
Other complication	92 (42)
**Location of death**	
Outside hospital	61 (22)
ED	74 (27)
Hospital (in-patient)	143 (51)
Missing	2 (—)
**Antiviral therapy received**	
Yes	112 (40)
Oseltamivir	104 (37)
Zanamivir	0 (—)
Peramivir	14 (5)
No	167 (60)
Unknown	1 (—)
**Duration of illness**	
Median days (range)	4 (2–10)
**Bacterial testing from sterile site performed**	
Yes	118 (55)
No	95 (45)
Unknown	67 (—)
**Bacteria isolated from sterile site^§§^**	
Yes	42 (41)
No	60 (59)
Unknown	16 (—)
**Bacteria isolated from sterile site^¶¶^**	
*Streptococcus pneumoniae*	8 (19)
*Staphylococcus aureus*, susceptibility not specified	6 (14)
Group A *Streptococcus*	6 (14)
MRSA	3 (7)
MSSA	1 (2)
Other	23 (55)
Influenza vaccination status***	
Fully vaccinated	22 (11)
Not fully vaccinated	186 (89)
Ineligible for vaccination (age <6 mos)	20 (—)
Missing	52 (—)

**Abbreviations**: ACIP = Advisory Committee on Immunization Practices; ED = emergency department; MRSA = methicillin-resistant *Staphylococcus aureus*; MSSA = methicillin-sensitive *Staphylococcus aureus*; PCR = polymerase chain reaction.

* Percentage calculated among 169 children with known influenza A subtype.

^†^ Percentage calculated among four children with known influenza B lineage.

^§^ Categorized as neurologic disorder (including moderate to severe developmental delay, seizure disorder, cerebral palsy, or neuromuscular disorder), pulmonary disease (including asthma/reactive airway disease, cystic fibrosis, or other chronic pulmonary disease), chromosome abnormality or genetic disorder, cardiac disease (including congenital heart disease), immunosuppressive condition (including cancer diagnosis or treatment during the previous 12 months), endocrine disorder (including diabetes mellitus), mitochondrial disorder, renal disease, pregnancy, and other medical conditions (including blood disorders, obesity, skin or soft tissue infections, or hepatic diseases).

^¶^ Calculated as the count of types of underlying medical conditions, including neurologic disorder, pulmonary disease, chromosome/genetic disorder, congenital heart disease or other cardiac disease, immunosuppressive condition, endocrine disorder, obesity, mitochondrial disorder, renal disease, and pregnancy. Percentage calculated among 148 children with underlying medical conditions.

** Percentage calculated among 262 children with known medical history.

^††^ Percentage calculated among 218 children with complications reported during acute illness.

^§§^ Percentage calculated among 102 children with a specimen collected for bacterial culture from a normally sterile site with known results.

^¶¶^ Percentage calculated among 42 children with bacteria cultured from a sterile site.

*** Children aged ≥6 months–8 years were considered fully vaccinated if they received an influenza vaccine during the current influenza season (≥14 days before illness onset) and at least two total influenza vaccines (either 2 doses during the current season, or 1 dose during the current season and 1 dose during a previous season). Children aged 9–17 years were considered fully vaccinated if an influenza vaccine dose was received during the current season and ≥14 days before illness onset.

### Clinical Characteristics and Influenza Vaccination Status

Among 262 pediatric decedents with available information on medical history, 148 (56%) had at least one reported underlying medical condition.^¶^ Among these, a neurologic condition was reported most frequently (93; 63%); among children with neurologic conditions, approximately two thirds (59; 63%) were described as having developmental delay.

Among 260 decedents who were age-eligible for vaccination, sufficient information to determine vaccination status was available for 208 (80%). Among those with known vaccination status who were vaccine-eligible, 186 (89%) had not been fully vaccinated against influenza during the 2024–25 season. Although influenza vaccination coverage was low overall, the percentage of children who were not fully vaccinated was slightly lower among children with medical conditions (86%) than among those without (95%).

### Clinical Course and Location of Death

Clinical complications before death were documented for 218 (88%) of 247 children with available data. Among the 247 children for whom data were available, the most common complication experienced before death was shock or sepsis (108; 50%) followed by pneumonia (82; 38%), acute respiratory distress syndrome (60; 28%), seizures (53; 24%), and encephalopathy or encephalitis (40; 18%). Isolation of a bacterial pathogen from a sterile site was reported for 42 (41%) of 102 children who received testing. The most commonly isolated pathogens were *Staphylococcus aureus*, *Streptococcus pneumoniae,* and group A *Streptococcus*. Overall, 112 (40%) children were treated with influenza antiviral medications, most commonly oseltamivir (104; 93%).

Among 278 deaths with information on location of death, 61 (22%) occurred outside a hospital, 74 (27%) occurred in an emergency department (ED), and 143 (51%) occurred in a hospital after admission (SupplementaryTable). The median interval from illness onset to death among children who died outside a hospital, in an ED, and while hospitalized was 3 days (IQR = 1–6 days), 2 days (1–4), and 7 days (4–13), respectively. The median number of days from symptom onset to death was 4 days (IQR = 2–10 days). Among children who died outside a hospital, in an ED, and in a hospital, influenza antivirals were received by 23%, 11%, and 62%, respectively.

## Discussion

The 2024–25 influenza season was marked by the highest number of pediatric deaths since influenza-associated pediatric mortality became nationally notifiable in 2004 (excluding the 2009–10 influenza A(H1N1)pmd09 pandemic, during which the overall highest number of pediatric deaths [288] occurred). Previously, the highest number of deaths reported during a nonpandemic influenza season was 210 during the 2023–24 influenza season. The lowest number of influenza-associated pediatric deaths occurred during the 2020–21 season, immediately after the start of the COVID-19 pandemic, when influenza virus circulation plummeted; during that season, only a single influenza death in a child was reported. Increasing numbers of deaths have been reported in each subsequent season since 2020–21.

According to a preliminary assessment, the 2024–25 influenza season has been associated with at least 43 million illnesses, 560,000 hospitalizations, and 38,000 deaths, and was the first high-severity season since the 2017–18 season. High severity was observed across all age groups. Influenza seasons are categorized as low, medium, or high severity in assessments conducted by CDC that incorporate three indicators: 1) the percentage of influenza-like illness among all outpatient or ED visits; 2) the influenza-related hospitalization rate, and 3) the percentage of deaths attributed to influenza among all deaths (*3*).

Reasons for the increase in influenza activity during the 2024–25 season, including pediatric deaths, are not clear. Prevention efforts during the early years of the COVID-19 pandemic suppressed influenza activity and deaths (*4*), and as restrictions were lifted, influenza circulation during subsequent seasons resumed. Co-circulation of multiple influenza A virus subtypes (influenza A[H1N1]pdm09 and A(H3N2) with nearly equal distribution) might have led to increased influenza activity. These subtypes can each result in varying impacts and severity among different age groups (*5*).

Characteristics of pediatric deaths reported during the 2024–25 season were mostly consistent with deaths reported during previous seasons. In all but two seasons since surveillance began (i.e., during the 2012–13 and 2019–20 seasons), influenza A viruses have been associated with more pediatric deaths than have influenza B viruses. During the 2024–25 season, 56% of children who died had conditions associated with higher risk for severe illness; this percentage has ranged from 38% during the 2006–07 season to 69% during the 2009–10 season (FluViewInteractive|CDC). Whereas approximately 80% of pediatric decedents who were vaccine-eligible had not received seasonal influenza vaccine in previous seasons (*6*,*7*), during the 2024–25 season, approximately 90% of eligible children with known vaccination status who died from influenza were not fully vaccinated.

Approximately one half of children who died had not been admitted to a hospital at the time of death. Among children who died in an ED or another location outside a hospital, the interval from symptom onset until death was substantially shorter (median = 2–3 days) than it was for those who died in a hospital (median = 7 days). Children who died in EDs or outside a hospital were less likely to have an underlying medical condition than did those who died after being hospitalized, and very few had been treated with antiviral medications. Parents, caregivers, and clinicians should be mindful of warningsignsofrespiratoryviruscomplications when children are ill and should seek immediate medical care for the child.

During the 2024–25 influenza season, the virus type and subtype distribution observed in pediatric mortality surveillance was similar to that from public health laboratory (PHL) surveillance, which monitors circulating viruses among a larger population. Influenza A viruses represented 86% of viruses detected in pediatric mortality and 89% among persons aged <25 years in PHL surveillance systems. Among pediatric deaths associated with influenza A viruses with known subtype, 56% were (H1N1)pdm09 and 43% were H3N2 viruses. A similar distribution of subtypes was observed among persons aged <25 years in PHL data (47% [H1N1]pdm09 and 53% H3N2).

### Limitations

The findings in this report are subject to at least three limitations. First, deaths are likely underreported because of factors including failure to identify or diagnose influenza, attributing death to another cause even if influenza was identified, and nonreporting. Thus, the number of reported cases likely represents an underestimate. Second, misclassification of underlying medical conditions, vaccination status, bacterial co-infections, and other characteristics of the children is possible. Misclassification might have been more likely among children for whom little clinical data were available because of young age, limited exposure to health care providers, or rapid progression from illness onset to death. Finally, data were missing from some reports for a number of variables, including medical conditions and complications. Data on antiviral treatment, medical conditions, and complications were more likely to be missing for children who died outside a hospital or in an ED than for those who died in a hospital.

### Implications for Public Health Practice

Influenza can cause serious illness and death in children; therefore, preventing infection, particularly among those who have underlying medical conditions, can reduce influenza-associated morbidity and mortality. All persons aged ≥6 months without a contraindication should receive an annual influenza vaccine; vaccinating children annually against influenza can help prevent severe illness and death.

## Footnotes

* Case report form includes information on demographic characteristics, medical history, and clinical information about the illness.

† Twenty children aged <6 months were ineligible for influenza vaccination. Children aged 6 months–8 years were considered fully vaccinated if they received an influenza vaccine during the current influenza season (≥14 days before illness onset) and at least two total influenza vaccines (either 2 doses during the current season, or 1 dose during the current season and 1 dose during a previous season). Children aged 9–17 years were considered fully vaccinated if an influenza vaccine dose was received during the current season and ≥14 days before illness onset.

§ 45 C.F.R. part 46.102(l)(2), 21 C.F.R. part 56; 42 U.S.C. Sect. 241(d); 5 U.S.C. Sect. 552a; 44 U.S.C. Sect. 3501 et seq.

¶ Conditions were categorized as neurologic disorder (including moderate to severe developmental delay, seizure disorder, cerebral palsy, or neuromuscular disorder), pulmonary disease (including asthma/reactive airway disease, cystic fibrosis, or other chronic pulmonary disease), chromosome abnormality or genetic disorder, cardiac disease (including congenital heart disease), immunosuppressive condition (including cancer diagnosis or treatment during the previous 12 months), endocrine disorder (including diabetes mellitus), mitochondrial disorder, renal disease, pregnancy, and other medical conditions (including blood disorders, obesity, skin or soft tissue infections, or hepatic diseases).
